# Agreement between Azure Kinect and Marker-Based Motion Analysis during Functional Movements: A Feasibility Study

**DOI:** 10.3390/s22249819

**Published:** 2022-12-14

**Authors:** Sungbae Jo, Sunmi Song, Junesun Kim, Changho Song

**Affiliations:** 1 Department of Physical Therapy, College of Health Science, Sahmyook University, Seoul 01795, Republic of Korea; 2 Rehabilitation Science Program, Department of Health Science, Graduate School, Korea University, Seoul 02841, Republic of Korea; 3 Department of Health and Environmental Science, College of Health Science, Korea University, Seoul 02841, Republic of Korea; 4 Department of Physical Therapy, College of Health Science, Korea University, Seoul 02841, Republic of Korea; 5 BK21FOUR Program: Learning Health Systems, College of Health Science, Korea University, Seoul 02841, Republic of Korea

**Keywords:** motion capture, activities of daily living, depth sensor, 3D motion analysis

## Abstract

(1) Background: The present study investigated the agreement between the Azure Kinect and marker-based motion analysis during functional movements. (2) Methods: Twelve healthy adults participated in this study and performed a total of six different tasks including front view squat, side view squat, forward reach, lateral reach, front view lunge, and side view lunge. Movement data were collected using an Azure Kinect and 12 infrared cameras while the participants performed the movements. The comparability between marker-based motion analysis and Azure Kinect was visualized using Bland–Altman plots and scatter plots. (3) Results: During the front view of squat motions, hip and knee joint angles showed moderate and high level of concurrent validity, respectively. The side view of squat motions showed moderate to good in the visible hip joint angles, whereas hidden hip joint angle showed poor concurrent validity. The knee joint angles showed variation between excellent and moderate concurrent validity depending on the visibility. The forward reach motions showed moderate concurrent validity for both shoulder angles, whereas the lateral reach motions showed excellent concurrent validity. During the front view of lunge motions, both the hip and knee joint angles showed moderate concurrent validity. The side view of lunge motions showed variations in concurrent validity, while the right hip joint angle showed good concurrent validity; the left hip joint showed poor concurrent validity. (4) Conclusions: The overall agreement between the Azure Kinect and marker-based motion analysis system was moderate to good when the body segments were visible to the Azure Kinect, yet the accuracy of tracking hidden body parts is still a concern.

## 1. Introduction

Accurate evaluation of movements during daily tasks is vital to provide efficient telehealth services, such as telerehabilitation and teleassessment. The marker-based motion analysis system is well-known as the gold standard tool for providing both qualitative and quantitative assessments of human motions [1,2]. The system provides an accurate and precise method to evaluate joint kinematics based on marker placement on anatomical landmarks and has been used in many fields, including sports [3] and rehabilitation [4]. However, marker-based motion analysis is not easily adaptable in most clinical settings because it requires enormous cost, time, space, and highly trained personnel [5]. To overcome these drawbacks, depth-sensor-based motion analysis has emerged rapidly.

One of the most well-known depth sensor devices, Kinect, was developed by Microsoft in 2010, which integrates RGB and infrared (IR) cameras to track human motions in 3D. The sensor uses the principle of structured light, in which IR radiation is projected sequentially to illuminate the scene in a dotted pattern. The IR camera then observes the dot pattern and estimates the depth information using triangulation. The system returns segmental coordinates that are tracked based on artificial intelligence (AI) trained on a large dataset of labeled depth images [6,7].

Kinect was originally designed to be used as a gaming controller that allowed users to engage in Xbox games without using a control device. The most recently developed version of the depth sensor, the Azure Kinect, provides an accurate and precise measure of human motion [8,9]. Azure Kinect utilizes the time-of-flight (TOF) principle, which calculates distance by the time of emitted light to reach the object and return to the camera [10,11]. Azure Kinect is cheap, noninvasive, and provides data simultaneously and thereby can be easily set up in a clinical environment. Many studies have used sensors to analyze gait [7], posture [12,13], and motion [14] during activities and exercises. 

Guess et al. [15] compared the sensor with marker-based motion analysis during gait parameter measurements and found a strong correlation between the two for all spatiotemporal parameters. Thomas et al. [16] used the sensor to analyze the five-times-sit-to-stand test, which is one of the fundamental assessments to evaluate the normal physical function of a person. They found that the results were very promising when compared with marker-based motion analysis. They were also able to distinguish four distinct phases of sit-to-stand motion using the sensor. In another study, Azure Kinect was used to assess shoulder range of motion [17]. They also found high reliability for all shoulder motion ranges. Azure Kinect also has its advantage in unobtrusive monitoring of subjects during rehabilitation programs [18] or, furthermore, in telerehabilitation [19]. Chen acknowledged that “as depth-sensor-based motion analysis has emerged, wearable sensors will not be required in the future” [20]. 

Functional movements are the movements based on real-life situations that are required to perform everyday tasks [21]. It is the ability to perform and maintain efficient and accurate movement patterns that are fundamental to daily life [22]. The ability to perform functional movements is indispensable because it can prevent injuries [23] and pain [24]. To achieve functional movement, muscle strength, endurance, coordination, flexibility, balance, and movement efficiency are imperative [18]. Squats are an example of a functional movement in which humans adapt variations to accomplish tasks of activities of daily living [25]. Squats can also be used as an essential functional training because they recruit multiple muscle groups during a single movement [23,24]. Lunge is also regarded as a functional activity and is used to assess hip muscle and knee performances [26,27,28]. Functional shoulder movements are also important during activities of daily living, such as preparing meals and household chores [29]. Assessing functional shoulder movements involves reaching objects in various directions [30]. However, to the best of our knowledge, functional movements have not been assessed using the Azure Kinect.

In most of the aforementioned studies, depth sensors were located in front of the subjects. At this angle, the camera detects frontal plane movements using IR and RGB tracking methods, while sagittal movements are estimated using depth information. However, variations can occur depending on the perspective of the sensor [31]. A previous study confirmed significant interaction effects between measurement angles during gait analysis using the Azure Kinect [31]. The study mentioned that Azure Kinect tracked hip and knee joint angles better in the sagittal plane. Furthermore, many of the preceding studies have not accounted for interference between Azure Kinect and retro-reflective markers during their validation procedures [15,16,32]. The interference effect had been reported previously [17,31,33,34]; thus, studies that validate the use of Azure Kinect should provide efforts to overcome this effect.

The purposes of this study were to (1) provide validity of the Azure Kinect by comparing it with the gold standard marker-based motion analysis system, (2) compare the accuracy of hidden body parts during the side view of squat and lunge, and (3) address the measurement strategy to overcome interference effect between Azure Kinect and marker-based motion capture. We hypothesized that the Azure Kinect and marker-based motion analysis would show high levels of correlation. We also hypothesized that the hidden body parts would show lower correlation compared to the visible body parts.

## 2. Materials and Methods

### 2.1. Participants

The current study recruited healthy adults from ‘S’ university located in Seoul, Republic of Korea. The inclusion criteria were as follows: subjects without (1) orthopedic injuries, (2) neurological disease, (3) pain or inflammation, and (4) visual or hearing problems. From 30 volunteers, a total of 12 healthy volunteers were included in the study, including six males and six females who met the inclusion criteria. The purpose and procedures of the study were explained to all the participants, and those who agreed to participate provided written consent. This study was approved by the institutional review board of Sahmyook University (SYU 2022-06-003-001). 

### 2.2. Experimental Procedures

**Figure 1:**
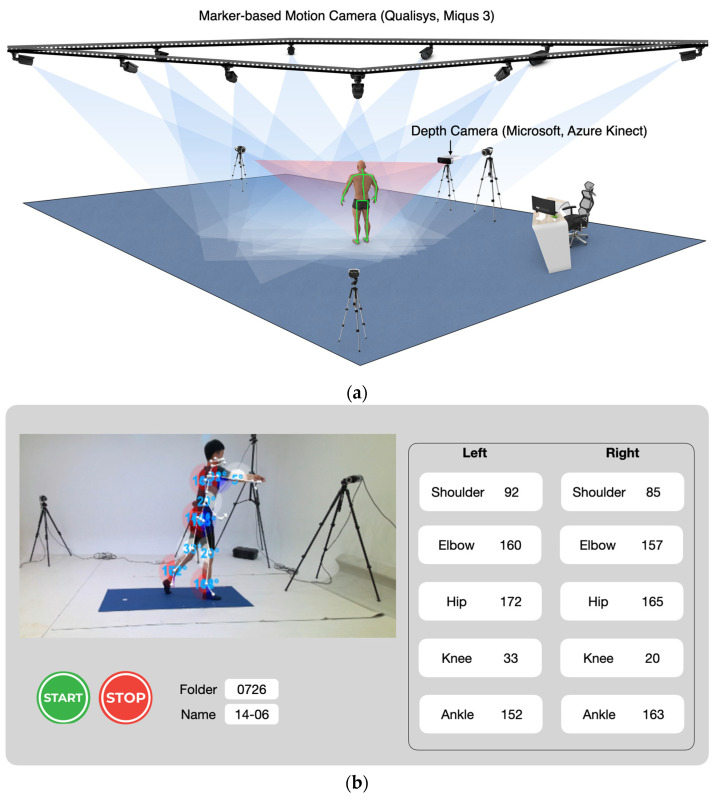
(**a**) Overview of experimental settings; (**b**) tracking of the movements using the developed program.

All experimental procedures were performed at a motion analysis laboratory located in ‘S’ university in Seoul, Republic of Korea. The current study included six tasks that are components of activities of daily living: front view squat, side view squat, reach forward, reach to side, front view lunge, and side view lunge. The side views of forward reach and lateral reach were excluded from this study, as results from our pilot study showed that side view of forward reach and side view of lateral reach displayed very similar results with front view of lateral reach and front view of forward reach respectively, hence the data were mutually replaceable. Movement data were collected using a depth camera (Azure Kinect, Microsoft, Redmond, WA, USA) and 12 infrared cameras (Miqus 3, Qualisys Ltd., Göteborg, Sweden) while the participants performed the movements (Figure 1a). Previous studies [15,16,32] reported the use of two cameras simultaneously; however, we noted that the accuracy of the depth camera dropped, possibly because of the markers reflecting the infrared from the cameras; therefore, we did not collect data simultaneously. A metronome and verbal queuing from an assessor were used to match the two trials: (1) five repetitions while the depth camera was captured, and (2) five repetitions while marker-based motion was captured. The data from the two systems were compared to confirm the validity of the depth camera.

### 2.3. Azure Kinect Data Collection

Kinematic data from the Azure Kinect were measured using a program built using Unity software (2021.3.11f1, Unity Technologies, San Francisco, USA). The program was built based on the Azure Kinect body tracking SDK (Azure Kinect SDK 1.3.0, Microsoft, Washington, USA) (Figure 1b). The program tracks and returns the 3D coordinates of the 32 markers to the CSV files. The shoulder, hip, and knee joints were calculated based on the movements of the coordinates. All the data were collected at 30 Hz. During the side view squat and lunge, the right side of the body was in front of the camera, consequently, the left side was hidden from the camera.

### 2.4. Calculations of Joint Angles from Azure Kinect Data

To calculate the joint angle based on the coordinate data from Azure Kinect, trigonometric functions were used in two different methods. The knee flexion and extension angles were calculated using the hip, knee, and ankle coordinates (formula (1)). A virtual vertical line perpendicular to the shoulder/hip coordinates was drawn, and the angle between the vertical line and the line between the shoulder-to-elbow/hip-to-knee coordinates was used to calculate shoulder/hip angles (formula (2)).

(1) $$\theta_{knee}=\tan^{-1}\left(\frac{y_{2}*\left(x_{1}-x_{3}\right)+y_{1}*\left(x_{3}-x_{2}\right)+y_{3}*\left(x_{2}-x_{1}\right)}{\left(x_{2}-x_{1}\right)*\left(x_{1}-x_{3}\right)+\left(y_{2}-y_{1}\right)*\left(y_{1}-y_{3}\right)}\right)$$

where $x_{1}$ and $y_{1}$ are the hip joints; $x_{2}$ and $y_{2}$ are the knee joints; and $x_{3}$ and $y_{3}$ are the ankle joints.

(2) $$\theta_{hip}=\tan^{-1}\left(\frac{\left(y_{1}-y_{2}\right)}{\left(x_{1}-x_{2}\right)}\right)$$

where $x_{1}$, $y_{1}$ is knee joint and $x_{2}$, $y_{2}$ is hip joint.

### 2.5. Three-Dimensional Motion Data Collection

Kinematic data from marker-based motion analysis was measured using 12 infrared cameras. A total of 63 reflective markers were used in the study, and anatomical markers were placed at the following anatomical landmarks: right and left acromion, shoulders at the acromion and greater tubercle lines, lateral and medial elbows, wrists, knees, and ankles, anterior superior iliac crests, posterior superior iliac crests, and highest points of iliac spines. These anatomical markers provided the coordinates of each marker, enabling the creation of a skeletal model with body segments. The tracking markers that tracked the motions of each body segment were placed on the following segments: the trunk, right and left upper arms, lower arms, thighs, and shanks. The tracked markers were labeled using a motion capture system (Qualisys Track Manager, Qualisys Ltd., Sweden), and the angles between each segment during movements were calculated using Visual 3D (v4.96.6, C-Motion, Boyds, MD, USA) software after creating a skeletal model for each participant using the software. All data were collected at 200 Hz. The biomechanical analysis was performed by selecting appropriate sequences for each joint, where the Visual 3D software automatically calculated angle values during the movements [35]. 

### 2.6. Data Analysis

The data from Azure Kinect and Visual 3D were exported to CSV files and then analyzed using MATLAB (MATLAB 2021a, Mathworks Inc., Natick, MA, USA). Signals were filtered with a fourth-order Butterworth filter at 6 Hz. The marker-based motion data were downsampled from 200 Hz to 30 Hz to synchronize with the data from the Azure Kinect.

### 2.7. Statistical Analyses

All statistical measures were calculated using MedCalc^®^ statistical software (v. 20.014, MedCalc^®^ Software Ltd., Ostend, Belgium). Concurrent validity Azure Kinect and marker-based motion analysis were measured using intraclass correlation coefficients (ICCs) with 95% confidence intervals (CIs). Correlation and ICC grading followed the guidelines: <0.50 = poor; 0.5–0.75 = moderate; 0.75–0.90 = good; >0.90 = excellent [36]. The comparability between marker-based motion analysis and Azure Kinect was visualized using Bland–Altman plots and scatter plots. Outliers were defined as 95% limits of agreement (mean difference ± 1.96 standard deviation). Statistical significance was set at *p* < 0.05.

## 3. Results

In the present study, a total of twelve healthy adults were recruited. The participants’ average age was 23.17 ± 1.95 years, with average height, weight, and BMI of 171.90 ± 8.52, 61.17 ± 12.81, and 20.53 ± 2.58, respectively.

Table 1 displays the R, R^2^, and ICC for the overall data between the Azure Kinect and the marker-based motion analysis. During the front view of squat motions, hip joint angles showed moderate concurrent validity (right hip, ICC 3.1 = 0.6302; left hip, ICC 3.1 = 0.5274), while knee joint angles showed a high level of concurrent validity (right knee, ICC 3.1 = 0.8989; left knee, ICC 3.1 = 0.9076). The side view of squat motions showed moderate to good concurrent validity for hip joint angles (right hip, ICC 3.1 = 0.8251; left hip, ICC 3.1 = 0.6378), while knee joint angles showed variation between excellent and moderate concurrent validity (right knee, ICC 3.1 = 0.9185; left knee, ICC 3.1 = 0.6625). The forward reach motions showed moderate concurrent validity for both shoulder angles (right shoulder, ICC 3.1 = 0.7068; left shoulder, ICC 3.1 = 0.7816), while the lateral reach motions showed excellent concurrent validity (right shoulder, ICC 3.1 = 0.9235; left shoulder, ICC 3.1 = 0.9069). During the front view of lunge motions, both hip and knee joint angles showed moderate concurrent validity (right hip, ICC 3.1 = 0.6929; left hip, ICC 3.1 = 0.5645; right knee, ICC 3.1 = 0.6821; left knee, ICC = 0.5826). The side view of lunge motions showed variations in concurrent validity, while the right hip joint angle showed good concurrent validity, and the left hip joint showed poor concurrent validity (right hip, ICC 3.1 = 0.7843; left hip, ICC 3.1 = 0.2868).

## 4. Discussion

**Figure 2:**
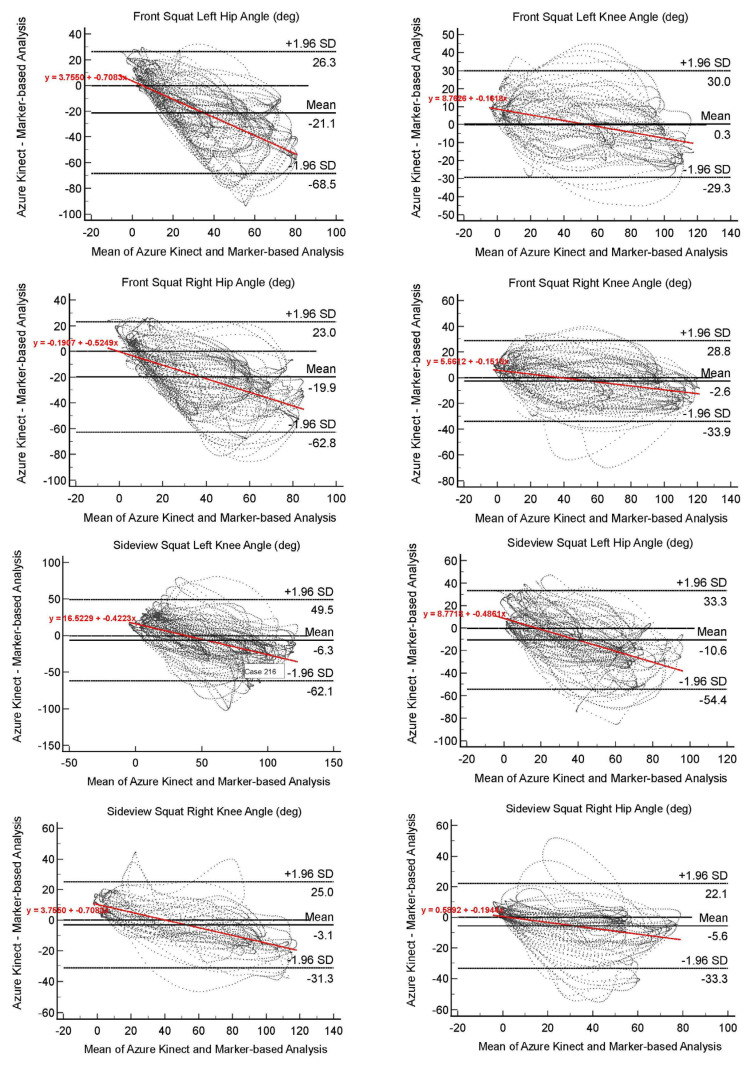

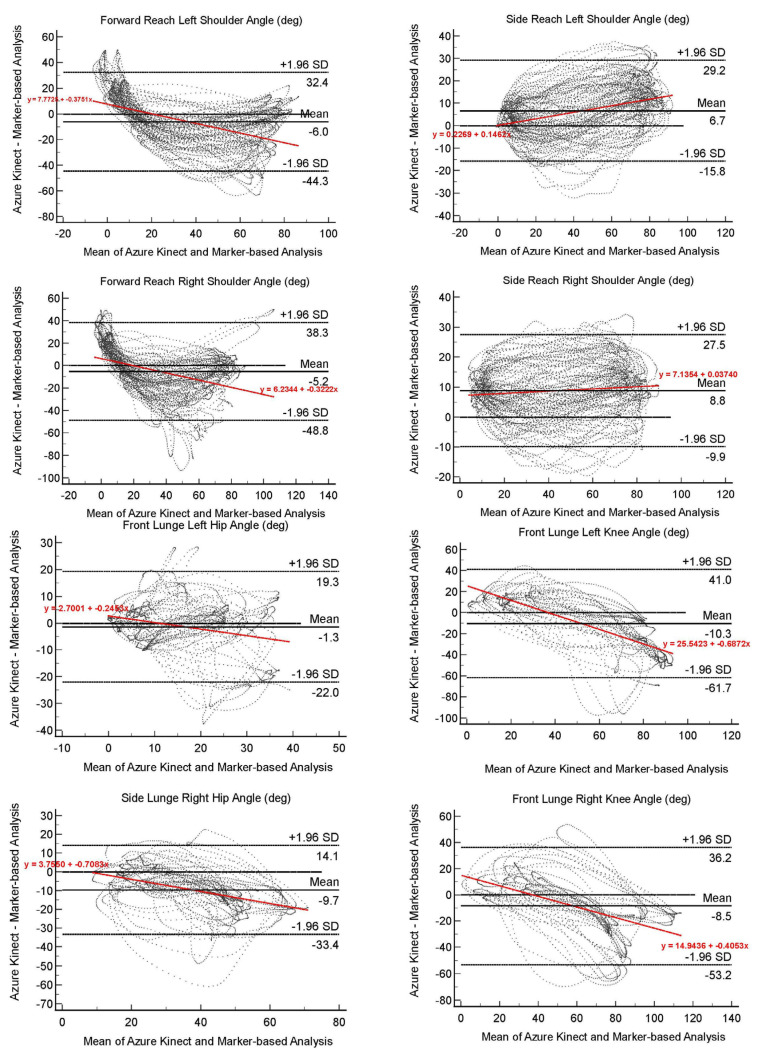

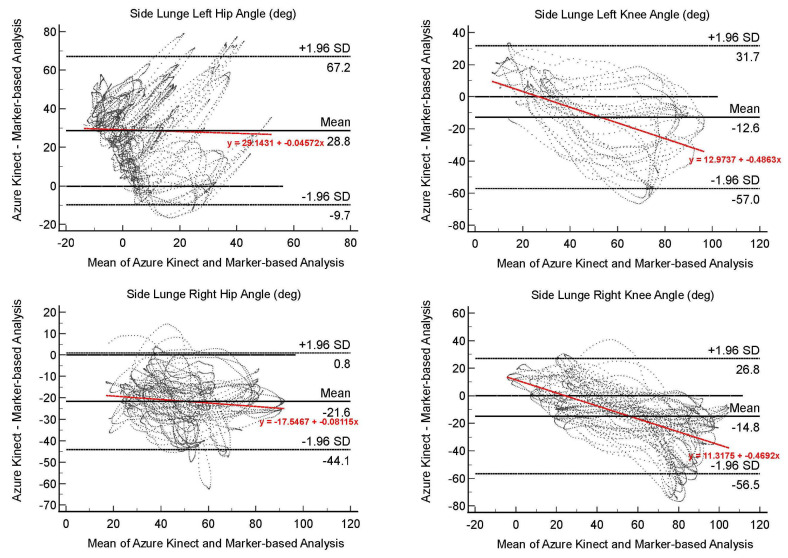
Bland–Altman plots to visualize agreement between Azure Kinect and marker-based motion analysis in each joint angle during six different functional movements for all participants.

The current study assessed the concurrent validity of the Azure Kinect, a well-known depth sensor, and marker-based motion analysis during functional daily movements, including squats, lunges, and upper arm reaches. According to the findings of the study, the agreement between the two measurements generally showed a moderate to good correlation. The results highlight the feasibility of using the Azure Kinect to assess the functional movements of daily activities. The Bland–Altman plots show overall agreement between the Azure Kinect and marker-based motion analysis in each joint angle during six different functional movements (Figure 2).

**Figure 3:**
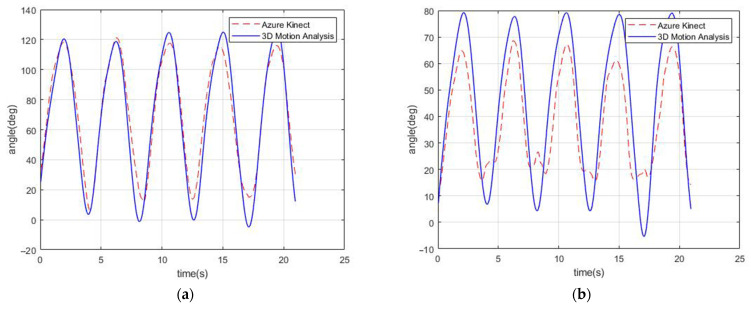
Example of participant 6 joint angle comparisons between Azure Kinect (front view) and marker-based motion analysis during squat movements. The starting and end points of the Azure Kinect and marker-based motion analysis were synced using Matlab software: (**a**) left knee angles show high agreement between the two systems; (**b**) inversions and greater gaps in the left hip angles observed due to errors in tracking of Azure Kinect.

The study results showed that knee motion when visible to the camera showed strong validity compared to hip motion (Figure 3). This is similar to the study by Thomas et al. [16], who also noted a greater estimation gap between Kinect data and marker-based motion data in the hip angles than in the knee angles. The strong agreement during tracking of the knee angles may suggest the reliability of using the Kinect during gait analysis, as well as clinical measures such as five-times-sit-to-stand [16], one-leg standing [37], and timed up-and-go test [38]. The variations in accuracy between hip and knee angles might be since Azure Kinect only adopts SDK algorithms to track the center of each joint, where the 3D analysis system tracks anatomical reflective markers that provide precise coordinates. However, we believe that future versions may adapt to a better precision in tracking the hip joint and overcome these errors.

Ankle kinematics are also important factors for analyzing gait and functional movements. However, during a pilot study prior to the current study, we identified that the Azure Kinect shows very poor tracking ability of the feet; therefore, data from foot coordinates were excluded from this study. A recent study noted that the Kinect displayed a discrepancy greater than 50 mm in the spatial agreement of ankle/foot markers [7]. This is somewhat different from the study of Guess et al. [15], who measured spatiotemporal parameters of gait using Azure Kinect. While the depth sensor relies heavily on depth measurements, the ground may affect the tracking of the ankle and foot joint centers, especially when the foot is in contact with the ground. Consequently, as suggested by Yeung et al. [31], a calibration method may be required to differentiate between the foot and ground during the tracking of the ankle and foot joint centers. Another solution is to use multiple depth cameras at different viewing angles and synchronize the data from the cameras.

The Azure body tracking SDK is known to estimate the coordinates of hidden extremities based on the artificial intelligence learned from decision forests trained using a large dataset. During the side view of the squat and lunge movements, the left leg was hidden behind the other leg. According to the results of our study, the estimation of hidden body parts was not accurate. As squat movements were viewed from the side direction, the concurrent validity of the left hip angle was 0.6378 compared to that of the visible leg, which was 0.8122. Knee angles also presented a similar phenomenon, but with an even greater difference, as the visible knee displayed an ICC of 0.9185, while the other knee displayed an ICC of 0.6625. These findings are rare, as most studies assess gait, which demonstrates alternative movement of the two legs, allowing the depth sensor to track both legs with minimal overlapping period [7,15,16,31]. However, the squat at the side-view angle from the camera makes the overlapping of the legs continuous. The front and side view lunge motion results also displayed notable discrepancies between the two legs. To perform lunges, one leg remains in front, while the other leg moves backward. In the front view, the front hip and knee showed ICC values of 0.6929 and 0.6821, while the back hip and knee showed ICC values of 0.5645 and 0.5826, respectively. Although both legs showed moderate agreement, the back leg was slightly lower. In the side view from the Azure Kinect, the hip extension angle of the back leg was not properly tracked. In comparison with the front hip and knee joint and back knee joint angles showing moderate-to-good agreement with the marker-based motion analysis, the hip joint of the left leg showed poor agreement. It appeared that the depth information of the left hip joint extension was not trackable. This may be due to the invisibility of the back hip joint center, causing errors during hip angle tracking. This may suggest using the frontal view angle of the Kinect to track alternative movements of the legs, such as the lunge and gait. To the best of our knowledge, this study is the first to validate the hidden body segments from the viewing angle of the Azure Kinect with marker-based motion analysis.

The ability to track the upper and lower extremities was not significantly different between the groups. A previous study by Ozsoy et al. [17] assessed shoulder range of motion and showed good agreement between the Azure and 3D analysis (shoulder flexion, ICC = 0.82), while Thomas et al. also found good agreement in hip flexion angles (left hip flexion, R^2^ = 0.907; right hip flexion, R^2^ = 0.921). 

Known interfering effects of the reflective markers on tracking the Azure Kinect coordinates were also observed during our pilot trials [17,31,33,39]. Therefore, the current study included two independent trials, each measuring the movements with the Azure Kinect first, and then the marker-based motion analysis. To overcome possible bias due to non-simultaneous measurements, all participants practiced all movements using a metronome rhythm before the measurements. In addition, the starting points of the Azure Kinect kinematic and marker-based motion analysis data were synced using the MATLAB software (Figure 2).

This study has several limitations. The Azure Kinect has a limited sample rate of 30 Hz, whereas the marker-based motion analysis system can capture more than 200 Hz. We downsampled the marker-based motion analysis data to synchronize the two datasets. Even human locomotion generally involves less than 10 Hz of voluntary frequency [40]; a low sample rate may limit precise tracking during a faster motion. The tracking errors of the hip joints, hidden legs, and hip extensions mentioned above are considerable limitations that might need to be overcome in future studies. In addition, a small number of homogeneous participants limit the usability of Azure Kinect in a variety of populations. Future studies may involve a larger number of participants with different groups such as subjects with locomotor dysfunctions. Despite these limitations, the results of the current study showed the feasibility of using the Azure Kinect to assess functional movements.

## 5. Conclusions

The current study confirmed the validity and feasibility of the Azure Kinect assessment of functional movements by comparing it with the gold standard marker-based motion analysis system. The variation depending on the measurement angle of the Azure Kinect was insignificant; however, the accuracy of the hidden leg in the side view decreased significantly.

## Figures and Tables

**Table 1:** Agreement between the Azure Kinect and marker-based motion analysis (*n* = 12).

Tasks	Analyzed Joint	R	R^2^	ICC	95% CI
Front view squat	Right hip	0.6972	0.4861	0.6302	0.6149 to 0.6450
	Left hip	0.6271	0.3933	0.5274	0.5096 to 0.5448
	Right knee	0.9083	0.825	0.8989	0.8938 to 0.9037
	Left knee	0.9186	0.8438	0.9076	0.9024 to 0.9126
Side view squat	Right hip (visible leg)	0.8251	0.6808	0.8122	0.8019 to 0.8220
	Left hip (hidden leg)	0.6953	0.4834	0.6378	0.6227 to 0.6525
	Right knee (visible leg)	0.9470	0.8968	0.9185	0.9132 to 0.9235
	Left knee (hidden leg)	0.7076	0.5007	0.6625	0.6490 to 0.6757
Forward reach	Right shoulder	0.7068	0.4996	0.6804	0.6674 to 0.6931
	Left shoulder	0.7816	0.6109	0.7389	0.7282 to 0.7492
Lateral reach	Right shoulder	0.9241	0.854	0.9235	0.9201 to 0.9266
	Left shoulder	0.9159	0.8389	0.9069	0.9029 to 0.9108
Front view lunge	Right hip (front leg)	0.7198	0.5181	0.6929	0.6757 to 0.7092
	Left hip	0.5752	0.3309	0.5645	0.5416 to 0.5865
	Right knee (front leg)	0.7256	0.5265	0.6821	0.6634 to 0.6999
	Left knee	0.6943	0.4821	0.5826	0.5592 to 0.6052
Side view lunge	Right hip (front leg)	0.7863	0.6183	0.7843	0.7727 to 0.7953
	Left hip	0.2869	0.0823	0.2868	0.2620 to 0.3112
	Right knee (front leg)	0.8009	0.6414	0.7318	0.7178 to 0.7452
	Left knee	0.7722	0.5963	0.6987	0.6807 to 0.7159

R, correlation; R^2^, coefficient of determination; ICC, intraclass coefficient (3,1); 95% CI, 95% confidence interval.

## Data Availability

The data presented in this study are available on request from the corresponding author.
